# 

**Published:** 1862-07

**Authors:** 


### BOOKS RECEIVED.

*On Military and Camp Hospitals, and the Health of Troops in the Field; being the results of a Commission to Inspect the Sanitary Arrangements of the French Army, and incidentally of other armies in the Crimean War. By* L. BAUDENS, *Inspector and Member of the Council of Health of the French Armies, formerly Surgeon-in-Chief, and first Professor of the Perfecting School of Val de-Grace, etc., etc. Translated and Annotated by* FRANKLIN B. HOUGH, M. D., *late an Inspector of the U. S. Sanitary Commission. New York:* BAILLIERE BROTHERS, 440 *Broadway*, 1862.

*Advice to a Mother on the Management of her Offspring. By* PYE HENRY CHAVASSE, *Fellow of the Royal College of Surgeons of England; formerly President of Queen's College Medico-Chirurgical Society Birmingham; author of "Advice to a Wife on the Management of her own Health." Reprinted from the Sixth London Edition. New York:* BAILLIERE BROTHERS, *Publishers*, 440 *Broadway*, 1862.

*Medico-Legal Contributions on Arsenic; containing Reports of a number of cases of Arsenical Poisoning, together with an account of the Methods employed in their Chemical Examination. By* CHARLES H. PORTER, M. D., *Professor of Chemistry and Medical Jurisprudence, Albany Medical College, Albany, N. Y. Albany:* CHARLES VAN BENTHUYSEN, 1862.

*The American Journal of Ophthalmology.— Vol.* 1, *No.* 1. JULIUS HOMBERGER, M. D., *Editor and Proprietor*, 24 *West* 12*th Street, New York. New York, July*, 1862: BAILLIERE BROTHERS, 440 *Broadway. Price*, $2 *per annum, in advance.*

*Thirty-Eighth Annual Register of the Rensselaer Polytechnic Institute in the City of Troy. July*, 1861.

*Wounded Trachea; Suffocation and Death in nineteen months. Suffocation by Plugging of the Trachea, by a portion of a diseased Bronchial Gland. By* J. R. BOULWARE, M. D., *of Albany. York. Albany:* CHAS. VAN BENTHUYSEN, 1862.

*Catalogue of Surgical Instruments. By* WADE & FORD, *Instrument Makers to the New York, Bellevue and City Hospitals. Manufacturers and importers of all ktnds of Surgical Instruments, Trusses, Syringes and Fine Cutlery, No.* 85 *Fulton Street, New York.*

All the different Surgical Instruments are represented or described and the prices given. Different cases of instruments are also fully described and the prices appended. Many physicians will be glad to know where they can obtain Fracture Apparatus, and the various splints for treating deformities, as Wood's, Sayer's or Buck's. This Catalogue of Instruments and prices can be obtained gratuitously by addressing WADE & FORD, 85 Fulton Street, New York City.

*The Atlantic Monthly for July.*—This number begins a new volume, and the publishers promise to make the Magazine more interesting in the future, and more valuable than it has ever been in the past. From their well known character and ability, we are willing to endorse their promise, and assure our friends and the public, that they will accomplish what they undertake.

*Harper's New Monthly Magazine for July. Published by* HARPER & BROTHERS, *Franklin Square, New York.*

*Peterson's Ladies' National Magazine for July. Published by* CHARLES J. PETERSON, 306 *Chesnut Street, Philadelphia.*

### INDEX.

Abortion, procuring of, ... 141
" induction of. ... 169
Aconite, tinc. of—poisoning. By W. Noble, M. D. ... 147
Address, before the Erie County Medical Society, By Prof. Austin Flint, 1
" introductory to the term of lectures in the University of Buffalo, By Prof. J. R. Lothrop, ... 129
" delivered before the Erie County Medical Society, By Henry Nichell, M. D. ... 193, 225
" before the Buffalo Med. Association, by C. C. F. Gay, M. D. 294
Amputation—Report of Sanitary Commission, ... 272
" of os humeri, By C. Winne, M. D. ... 235, 271
" through shoulder-joint, By C. Winne, M. D. ... 300
" cervix uteri, ... 346
Anaesthesia in puerperal convulsions. By P. S. Dorland, M. D. ... 269
Anaesthetic, Kerosolene ... 86
" ether, as an ... 151
" chloroform and ether, ... 276
Aneurism, aortic, ... 175
" traumatic, ... 321
Anus, imperforate, ... 268
Apoplexy, ... 139
Army, Buffalo physicians in the, ... 31, 320, 350
" information for persons desirous of entering, ... 123
" re-organization of the medical dep. in the, ... 180
" appointments in the, ... 199
" burial of soldiers, ... 319
" surgeon-general of the. ... 319
Bark and Iron Elixir of ... 349
Bibliographical Notices—Maxson's Practice of Medicine, ... 61
Bumstead on Venereal Diseases, ... 87
Barwell on Diseases of Joints, ... 90
Bedford on Principles and Practice of Obstetrics, ... 158
Taylor's Medical Jurisprudence, ... 159
Physicians' and Dentists' Pocket Memorandum, ... 158
West on Diseases of Women, ... 190
O'Reilly on the Placenta and Nervous System, ... 222
Gross' System of Surgery, ... 252
McLeod's Surgery in the Crimea, ... 253
Braithwaite's Retrospect, ... 253
Border Lines of Knowledge, By O. W. Holmes, M. D. ... 281

Bibliographical Notices,—Bedford on Diseases of Women and Children, 284
Commentaries on the Surgery of War, By G. J. Guthrie, M. D. 285
Bedford on the Principles and Practice of Obstetrics, 2d ed. 314
Gray's Descriptive and Surgical Anatomy, 315
Book about doctors, Dy J. Cordy Jefferson, 316
Sargent's Minor Surgery, 344
Health, By John Brown, M. D. 346
Barclay on Medical Diagnosis,
Williams on Diseases of the Eye,
Hand-book of Surgical Operations, By Stephen Smith, M. D. 377
Advice to Mothers, By Pye Henry Chavasse, 369
Medical Diagnosis, By A. W. Barclay, M. D. 378
Brain, obscure disease of 330
Brigade Surgeons, Homœpathic, 94
Bright's disease—Per-sulphate of iron in, 104
" Tanin, in 105
Buffalo Medical Association, Proceedings of, 12, 49, 72, 103, 139, 164, 203, 232, 264, 293, 325, 355,
Calculus, salivary, 103
Caries and necrosis of the humerus, 271
Cerebral disease following injury, 208
Charm doctors, 121
Charge against the Surgeon of the 1st Vt. Regiment, 31
Cholera infantum, By Henry Nichell, M. D. 193, 225
Clinical lecture on coition, 96
Chloroform in Puerperal Convulsions, By A. Wharton, M. D. 213
Chloroform and Ether 276
Club foot, By J. F. Miner, M. D. 321
Commencement of the University of Buffalo, 245
Copper, in the treatment of Taemia, 120
Croup—tracheotomy, By J. F. Miner, M. D. 321
Cystorrhœa vs matrimony, 121
Craig's Microscope, 380
Deaths, monthly record of, 28, 64, 128, 160, 192, 224, 256, 288, 352
Diabetes, 355
Diphtheria, 185
Dislocation of humerus, with fracture of surgical neck, By C. C. F. Gay, M. D. 79
Eclampsia, puerperal, 232
Editorial—Introductory, 28
Civil and Military Surgery, 57
New anæsthetic agent—Kerosolene, 66
Homœphathic Brigade Surgeons, 94
Medical lecture season, 185
Commencement of University of Buffalo, 245
Prof. E. K. Sanborne, 348
Buffalo Medical and Surgical Journal, 370
Death Certificates, 372
Epilepsy, 140
Erie County Medical Society, meeting of the 60, 359

Erysipelas—traumatic, ........ 12
" idiopathic, ........ 17
" phlegmonous, ........ 17
Ether, as an anæsthetic, ........ 73
Fistula, perineal, ........ 292
Fracture—treatment of, ........ 178
" starch bandages, in recent ........ 188
" of the skull, ........ 292
Gangrene, dry, By A. P. Philips, M. D. ........ 353
Heart disease, hypodermic injections in, By S. Barrett, M. D. ........ 203
History of the Origin and Transactions of the Medical Societies of Buffalo, By Thos. F. Rochester, M. D. ........ 33, 65, 97
Humerus—dislocation in the axilla, with fracture of surgical neck, ........ 79
" Osteo-sarcoma, necessitating amputation, By C. Winne, M. D. 235
" Amputation of the, By C. Winne, M. D. ........ 271
Hydrops, articuli, By S. Barrett, M. D. ........ 329
Introductory, ........ 29
Induction of premature labor, ........ 137
Joints opening into, ........ 266
Jaundice—artificial, ........ 369
Kerosolene, ........ 86
Lead in the treatment of Phthisis, ........ 110
Letter from—Charles Wilcox, M. D. ........ 26
" J. A. Peters, M. D. ........ 55
" Naval Hospital, Brooklyn, ........ 91
" Clarksburg, Va. ........ 113
" A New England physician, ........ 313
" W. H. Butler, M. D. ........ 374
Liver, abscess, in the ........ 165
Leucorrhœa and ulceration of cervix uteri during pregnancy, ........ 250
Lupus, ........ 260
Luxation of hip joint, spontaneous, ........ 50
Meatus auditorius extermus—larvæ of the common fly in the ........ 360
Muscles, action of the voluntary, By M. Mackell, M. D. ........ 307, 335
Mercury in the treatment of primary syphilis, By L. Parker, M. D. ........ 250
New Year, ........ 192
Notices—medical, ........ 128
Notes—surgical, By J. F. Miner, M. D. ........
Paralysis of the muscles of the eye and face from concussion, 257
Encephaloid tumor, ........ 259
Lupus, extirpation of the globe of the eye, ........ 260
Varicose veins, ........ 262
Stricture of the urethra, ........ 289
Croup—Tracheotomy, ........ 291
Depressed fracture of the skull, ........ 292
Traumatic aneurism, ........ 327
Club foot, ........ 323
Obituary, Wm. Treat, M. D. ........ 60
Bryant Burwell, M. D., ........ 214
Prof. E. K. Sanborne, ........ 341

Operation for removal of necrosed and carious bones,
By J. F. Miner, M. D,........ 148
Osteo-sarcoma of the humerus, by C. Winne, M. D.,........ 235
Paralysis of the eye and face,........ 257
" from gun shot wounds,........ 109
Per-chloride of iron in treatment of in-growing nails,........ 262
Per-sulphate of iron in, Bright's Disease,........ 104
Perineum, rupture of the,........ 166
Petroleum, By Prof. Geo. Hadley........ 161
Pneumonia, treatment of,........ 248
Poisoning, from rattle-snake bite, By L. S. Ham, M. D........ 82
" by tinc. aconite, By W. Noble, M. D.,........ 142
" Uræmic,........ 107
Polysarcia, By Ira T. Hopkins, M. D........ 114
Premature development........ 52
Prolapse of Funis, By G. N. Burwell, M. D........ 122
" " " Sanford Eastman, M. D........ 164
Postural treatment,........ 264
Puerperal convulsions treated with chloroform, by A. Wharton, M. D. 213
Rattle-snake bite, treatment of, By L. S. Ham, M. D........ 87
Rectum, stricture of the,........ 143
Report of the surgery after the battle of Bull's Run,........ 53
Selections, medical—Prolapse of Funis,........ 116
Expulsion of tæmia by copper,........ 120
Sickness in the Rebel army,........ 120
Cystorhoea vs. matrimony,........ 121
Charm doctors,........ 121
Aortic aneurism,........ 175
Conservative surgery,........ 176
Treatment of fractures,........ 178
Diphtheria,........ 185
Starch bandages in recent fractures,........ 188
Treatment of pneumonia,........ 248
Dr. L. Parker on the use of mercury in primary syphilis,........ 250
Leucorrhoea & ulceration of the cervix uteri during pregnancy, 251
Use of chloroform and ether,........ 276
Metropolitan health bill,........ 279
Extract from a letter to a New England physician........ 313
Artificial jaundice,........ 369
Larvæ common fly,........ 360
Induction of premature labor,........ 361
Drill for Auscultation,........ 363
Masturbation,........ 367
Iodide Potaissum,........ 368
Sciatica—hypodermic injections in,........ 305
Sick and wounded soldiers, provisions for,........ 311
Slowness of pulse without any well marked disease to account for it, 182
Societies—Buffalo Medical, 12, 49i 72, 103, 139, 164, 203, 232, 264
293, 325, 355,
" Erie County Medical,........60, 359

Societies—New York Medical, 237
" American Medical, 288, 319
St. Anthony's fire, 19
Stomach, spontaneous perforation of the, By W. H. Butler, M. D. 332
Surgery, civil and military, 59
Surgeon General of the U. S. Army, 319
Synovitis, rheumatic of the knee joint, 205
Tænia, expulsion of by copper, 120
Tannin, in bright's disease, 105
Tumor—aneurismal, of aorta, 175
" encephaloid, 259
" fibrous malignant,
Uraemiæ, 107
Urethra, stricture of, 289
Uterus, inversion of, By Prof. J. P. White, 22
" by Henry Nichell, M. D. 54
Occlusion of the 141
Vaccination of 21st Regiment, 80
Vaginitis and conjunctivitis, 52
Varicose veins, 262

